# Social Physique Anxiety Scale: Psychometric Evaluation and Development of a Chinese Adaptation

**DOI:** 10.3390/ijerph182010921

**Published:** 2021-10-17

**Authors:** Jiahui Jin, Sai-fu Fung

**Affiliations:** Department of Social and Behavioural Sciences, City University of Hong Kong, Hong Kong, China; jhjin2-c@my.cityu.edu.hk

**Keywords:** Chinese, student, dimensionality, SPAS, body image, anxiety

## Abstract

The Social Physique Anxiety Scale (SPAS) is a popular measure of individual anxiety related to body image. This study assessed the psychometric properties of the 12-, 9-, 8- and 7-item versions of the SPAS. Two cross-sectional studies recruited 466 Chinese university students. Study 1 (*n* = 273) evaluated the construct validity and internal consistency of the SPAS. Study 2 (*n* = 193) further assessed the construct validity, factorial validity, internal consistency, convergent validity and divergent validity of the SPAS. The results indicated that none of the existing SPAS versions possess good psychometric properties suitable for the Chinese student population. In short, a new 7-item version of the SPAS that is more suitable for measuring social physique anxiety among Chinese university students. The implications of our results and future research directions are discussed.

## 1. Introduction

Social physique anxiety (SPA) is the anxiety experienced when a person believes they are being observed or judged on their appearance; it is considered to be a subtype of social anxiety [1]. SPA manifests in an individual’s inability to view themselves as desirable to others. Crawford and Eklund [2] introduced self-presentation theory into the conceptual understanding of SPA. According to Goffman [3], anxious people always aim to make a good impression on others to seek positive evaluations, and SPA is the result of anxiety about self-presentation. Empirical research has suggested that people with a high level of SPA prefer settings that de-emphasise the physique rather than settings that emphasise the physique, which is aligned with the self-presentation explanation of SPA [2]. Studies have also found that more perfection-seeking people have higher SPA [4]. Hence, self-presentation and SPA are connected by the concept of perfection-seeking. The more strongly individuals seek to present a perfect image to others, the higher their SPA and the lower their body esteem [5,6].

Women were reported as having a higher level of SPA than men [5,7,8,9]. The limited research about the correlation between age and SPA showed the level of SPA grew with females’ age whereas decreased with males’ age with the participants aged from 11 to 24 [5], which means that college-aged women will have higher SPA level. The concept of SPA is highly overlapping with Body Image Dissatisfaction (BID) in the previous literature, with the goal of creating a good impression [10,11], which was found to be presented at higher levels in women as well [12]. Ålgars, Santtila [12] also reported females (aged 18 to 26) possessed the highest level of BID, which can be a reference for the characteristics of people with high SPA levels.

SPA was first proposed in a fitness context and has a strong connection with exercise [1,2,4,13]. The concept of SPA is therefore widely applied in the field of exercise psychology. People with a higher level of SPA are less likely to participate in exercise due to a fear of presenting themselves in front of others [2,13,14,15]. This observation is supported by Gammage, Ginis [16], who proposed a negative relationship between SPA and self-presentational exercise efficacy. Recent studies have also found that exercise may contribute to the mitigation of SPA [17]. This finding reflects the two approaches to cope with SPA proposed by Hart, Leary [1], which are avoidance and remedial behaviour. Avoidance is considered to be the primary behavioural tendency for coping with anxiety in general [18]. Remedial behaviour is regarded as a healthy behaviour to cope with SPA [19,20,21]. SPA also has a significant effect on dietary habits, which can influence physique presentation [7,22,23].

In addition to its physical effects, SPA influences the psychological dimension of self-esteem. Higher SPA is associated with lower self-esteem [24,25,26]. People with high levels of SPA also experience the emergence of social anxiety due to a lack of confidence in how the self presents to others [27,28]. Furthermore, a number of studies have reported that SPA reduces mental well-being and life satisfaction [29], and life satisfaction has been used as a measure of the impact of mental disorders [30]. Thus, it is possible to predict the impact of SPA on mental disorders, and relationships between SPA, analogue generalised anxiety disorder and analogue social anxiety disorder were confirmed [31].

The findings discussed above suggest that accurate measurement of SPA levels is of great significance in healthcare. To operationalise SPA, the Social Physique Anxiety Scale (SPAS) was originally proposed by Hart, Leary [1] in the context of gym fitness assessment. Although many studies have demonstrated satisfactory stability and validity of the 12-item unidimensional SPAS, its factor structure was much discussed. The plausibility of a second-order model was identified by scholars who argued that comfort with physique presentation (items 1, 2, 5, 8 and 11) and expectations of negative physical evaluation (items 3, 4, 6, 7, 9, 10 and 12) are factors subordinate to SPA [8,13,32]. As such, Eklund, Kelley [8] identified item 2 as problematic but did not modify it in their model. However, although the second-order model had a good model fit, it was eliminated as a methodological artefact without substantive meaning [33,34].

There was further controversy related to the item composition of the SPAS. Martin, Rejeski [33] extended Eklund, Kelley’s [8] view of item 2 and identified problems with items 1 and 5. They developed a 9-item unidimensional scale by excluding items 1, 2 and 5 and concluded that the unidimensional model was “more parsimonious and conceptually clear” than the two-factor model (p. 359). In a study on college students in America, Motl and Conroy [34] found that items 11 and 12 were problematic and proposed a 7-item unidimensional scale (items 3, 4, 6, 7, 8, 9, 10). This scale has been widely used in various countries [17,35,36,37,38]. Hagger, Aşçι [39] proposed an 8-item version after removing items 1, 5, 8 and 11, which is considered suitable for some European countries but remains controversial [40].

The development of an SPAS scale for the Chinese context is still in its initial stages. Isogai, Brewer [41] evaluated several scales on samples of female university students in Asian countries, including China, Japan, Korea and Thailand. A new 7-item scale (including items 3, 4, 6, 7, 9, 10 and 12) was found to improve the goodness-of-fit index in a Chinese sample [41]. However, despite the controversy related to the factor structure of the SPAS, there has been no further study to evaluate the SPAS scales in the Chinese context. To the best of our knowledge, there was also no empirical study to systematically evaluate the factor structure and psychometric properties of a Chinese version of the SPAS in separate male and female populations. In the present study, we also consider exploring a fresh version based on the results, in addition to evaluating existing models. The results of the present study provide a reliable and valid measurement of SPA that scholars and practitioners can use to evaluate SPA in Chinese societies.

## 2. Methods

### 2.1. Participants

We recruited 466 respondents in total for two cross-sectional studies. All questionnaires were completed by online university student groups in the social networking sites QQ, WeChat and Douban. There were two waves of data collection, in June and July 2021. The mean age of the respondents in Study 1 (*n* = 273) was 21.2 years (SD = 2.21); 25.6% were male and 74.4% were female. The mean age of the respondents in Study 2 (*n* = 193) was 23.5 years (SD = 0.87); 28.5% were male and 71.5% were female. Details of the participants’ demographic characteristics can be found in Table 1.

### 2.2. Measure

Study 1 used the original 12-item SPAS (SPAS-12) [1], which comprises 12 questions scored on a Likert-type scale ranging from 1 (definitely disagree) to 5 (definitely agree). The total scores range from 12 to 60. A higher score indicates greater appearance anxiety. Positively worded items (items 1, 2, 5, 8 and 11) are reverse-scored before summing. The internal consistency of the SPAS was reported to be 0.90, and the 8-week test–retest reliability was 0.82 [1]. Standard translation and back-translation procedures were applied to items that were initially translated into Chinese by a Chinese person who had lived in the UK for 4 years and then translated back into English by a Chinese person who had majored in English. Another fluent English and Chinese speaker compared the original English content with the translated content to overcome potential issues related to cross-cultural research and issues of equivalence [42,43,44]. An abbreviated 9-item unidimensional version of the SPAS (SPAS-9), which includes items 3, 4, 6, 7, 8, 9, 10, 11 and 12, was proposed by Martin, Rejeski [33]. The 8-item SPAS with a single-factor structure (SPAS-8) was proposed by Hagger, Aşçι [39] and includes items 2, 3, 4, 6, 7, 9, 10 and 12. Motl and Conroy [34] proposed a 7-item unidimensional version containing items 3, 4, 6, 7, 8, 9 and 10 (SPAS-7a). Isogai, Brewer [41] suggested an alternative version of the 7-item SPAS consisting of items 3, 4, 6, 7, 9, 10 and 12 (SPAS-7b). The questionnaire also included demographic questions, such as the sex and age of the respondent.

The questionnaire for Study 2 included all questions used in Study 1 and five construct-related scales to evaluate concurrent validity. The literature suggests that satisfaction with life [29], self-esteem [24,25,26], mental well-being [29] and self-efficacy [45,46] are negatively correlated with SPA and that social interaction anxiety is positively aligned with physique anxiety [27,28].

We used the Satisfaction with Life Index (SWL) [47], in which items are rated on a 7-point Likert-type scale ranging from 1 (strongly disagree) to 7 (strongly agree). The total scores range from 5 to 35, with a higher score indicating greater life satisfaction. Cronbach’s alpha for the scale was reported to be 0.81 by Diener, Emmons [47] and 0.89 in China by Kong, Zhao [48].

The Chinese version of the Rosenberg Self-esteem Scale (RSE) [49], which was validated by Wu, Zuo [50], was used to measure self-esteem. This scale includes 10 items that are rated on a 4-point Likert-type scale ranging from 1 (strongly disagree) to 4 (strongly agree). The total scores range from 10 to 40, with a higher score indicating greater self-esteem. Negatively worded items (items 2, 5, 6, 9) are reverse-scored before summing. The positive/negative attribution of item 8 is in dispute [50]. In this study, item 8 was regarded as positively worded, which is consistent with previous studies in China [50]. According to Song, Cai [51], the Cronbach’s alpha of this scale in Chinese university students was 0.83.

The participants’ mental health was assessed using the simplified Chinese version of the WHO (Five) Well-Being Index (WHO-5), which was released by the World Health Organization in 2007 [52,53,54]. This scale includes five items that are rated on a 6-point Likert-type scale ranging from 1 (at no time) to 6 (all of the time). The total scores range from 5 to 30, with a higher score indicating greater mental well-being.

The General Self-Efficacy Scale (GSE) [55] was translated by Zhang and Schwarzer [56]. This scale includes 10 items rated on a 4-point Likert-type scale ranging from 1 (absolutely incorrect) to 4 (absolutely correct). The total scores range from 10 to 40, with a higher score indicating greater self-efficacy. A recent study reported that the scale has good internal consistency in Chinese participants, with a Cronbach’s alpha of 0.91 [57].

The 6-item Social Interaction Anxiety Scale (SIAS-6) [58] was adopted to measure social interaction anxiety. The short version was chosen because the full version of the SIAS [59] has 20 questions, which may discourage participants. The translation was conducted by Peng, Lam [60]. This scale is rated on a 5-point Likert-type scale ranging from 1 (absolutely not me) to 5 (absolutely me). The total scores range from 10 to 40, with a higher score indicating greater social interaction anxiety. Fergus, Valentiner [58] reported that the shortened scale demonstrated good internal consistency, with a Cronbach’s alpha of 0.88.

### 2.3. Procedure and Data Analysis

To avoid potential translation-related problems, we conducted offline pilot studies involving 13 Chinese university students from a variety of disciplines, including mathematics, management, sociology and communication, and at different levels of education, including undergraduate and postgraduate. None of the participants in the pilot studies reported any problems understanding the translated version of the SPAS. The data from the pilot studies were excluded from the subsequent analysis.

The study was approved by the research ethics committee of the City University of Hong Kong. The participants provided informed consent, and all data collected were anonymous. The entire research process strictly adhered to relevant international ethical standards, such as the Declaration of Helsinki guidelines.

Confirmatory factor analysis (CFA) was used to evaluate the construct validity of the full and shortened versions of the scales. As the SPAS has Likert-type scale items with ordinal observed variables, a diagonally weighted least squares method was used to estimate parameters because it is less biased and more accurate than a maximum likelihood method [61]. The following criteria indicate good model fit: comparative fit index (CFI) > 0.95, Tucker–Lewis index (TLI) > 0.95, root mean square error of approximation (RMSEA) < 0.06, standardised root mean square residual (SRMR) <0.06 and χ^2^/df ≤ 3 [62,63,64,65,66,67]. To avoid the potential problem of overfitting [68], CFA and exploratory factor analysis (EFA) were conducted on different datasets. Data from Study 1 (*n* = 273) and combined data from Studies 1 and 2 (*n* = 466) were used to assess the construct validity of the SPAS via CFA as validation samples. To ensure that the findings did not differ across genders, CFA was also performed after separating the dataset according to gender.

EFA with principal axis factoring was used to evaluate the factorial validity of the 7-item SPAS we proposed, as the unusual performance of item 3 was found (with low factor loading). The Kaiser–Meyer–Olkin (KMO) test and Bartlett’s test of sphericity were used to assess whether the scales had good factor structures. According to Field [69], adequate factor structure criteria are as follows: KMO estimates > 0.70 and Bartlett’s test result that is significant at the *p* < 0.01 level. As the items are correlated with each other, the principal axis method with Promax was adopted [70,71]. According to Merenda [72], a threshold factor loading of 0.3 is the minimum when deciding to accept an item as belonging to a factor in social and behavioural science. The EFA was conducted on a calibration sample which is the data from Study 2 (*n* = 193).

The internal consistency of the shortened versions was assessed by using Cronbach’s alpha [73] and examining the corrected item-total correlation between items [74] with the data from Study 1 and Study 2, respectively. Recent validation literature recommends using McDonald’s omega to calculate reliability [75]. We therefore also calculated McDonald’s omega [76].

Concurrent validity was evaluated by other constructs related to SPA, as reported in previous studies. The SPA construct was shown to be significantly negatively correlated with satisfaction with life [29], self-efficacy [45,46], self-esteem [24,25,26] and mental well-being [29]. The literature also suggests that SPA is positively correlated with social interaction anxiety [27,28]. Hence, we used the SWL, RSE, WHO-5 and GSE to evaluate the divergent validity of the SPAS and the SIAS-6 to evaluate convergent validity based on the data from Study 2. These analyses were conducted using SPSS version 26.0 and R version 4.1.0 with the lavaan package version 0.6–8 [77].

## 3. Results

### 3.1. Construct Validity

Table 2 shows the CFA results for the five versions of SPAS proposed in previous studies. The results for SPAS-12 [1] and SPAS-9 [33] did not reach all of the cut-off values of the selected indexes in both Study 1 and the combined datasets. SPAS-8 [39], SPAS-7a [34] and SPAS-7b [41] fulfilled most of the cut-off criteria for a good model fit. However, item 3 had factor loadings below 0.3 in all of the above models and in both datasets, which is commonly regarded as an unacceptable value for factor loading [78,79]. For SPAS-8, the factor loadings for item 3 were 0.20–0.21, whereas the factor loadings for other items ranged from 0.58 to 0.87. For SPAS-7a, the factor loadings for item 3 ranged from 0.21 to 0.23, while other items’ factor loadings ranged from 0.71 to 0.86. For SPAS-7b, factor loadings for item 3 were 0.19–0.21, whereas those of other items ranged from 0.51 to 0.85.

Hence, a new version of SPAS is required. We performed CFA for SPAS-7a, SPAS-7b and SPAS-8, excluding item 3. The results for SPAS-6b (developed from SPAS-7b) and SPAS-7 (developed from SPAS-8) showed good model fit in both datasets, while SPAS-6a (developed from SPAS-7a) failed to reach the cut-off value of χ^2^/df with the combined data (χ^2^/df = 3.29). To eliminate differences between genders, we divided the combined data into male and female datasets and performed CFA with different gender groups. SPAS-6b failed to fulfil the criteria for good model fit in the female group, with χ^2^/df = 3.16. The results suggested that our newly proposed SPAS-7 (items 2, 4, 6, 7, 9, 10 and 12) showed the best fit index values for all indicators and with all datasets (see Table 3).

### 3.2. Factorial Validity

In the subsequent sections, we evaluate the factorial validity of the 7-item SPAS with items 2, 4, 6, 7, 9, 10 and 12 by EFA. The factor analysis results show KMO values and Bartlett’s test of sphericity for SPAS-7 of 0.889 (χ^2^ = 487.202, *p* < 0.001). The EFA result reveals that the new SPAS-7 has a single factor, and one factor extracted from the SPAS-7 explains 53.8% of the variance. The factor loadings for all items ranged from 0.58 to 0.80 (*n* = 193).

### 3.3. Internal Consistency

Descriptive statistics and correlations for the 7-item SPAS (*n* = 273 for Study 1; *n* = 193 for Study 2) are shown in Table 4. All seven items were normally distributed (skewness < 10; kurtosis < 3). All items were significantly correlated with each other (*p* < 0.01). The Cronbach’s alpha and McDonald’s omega results indicate that the SPAS-7 has good internal consistency (α = 0.86–0.89; ω = 0.86–0.89).

### 3.4. Convergent Validity

Table 5 shows that the new 7-item SPAS has good convergent validity, with a significant moderate correlation with SIAS-6 (α = 0.79; *r* = 0.55, *p* < 0.01), consistent with previous literature [27,28].

### 3.5. Divergent Validity

The results in Table 5 also show that the SPAS-7 possesses good divergent validity, in line with the results of previous research studies [24,29,45,46]. The 7-item SPAS was significantly correlated with the SWL (α = 0.88; *r* = −0.36, *p* < 0.01), RSE (α = 0.86; *r* = −0.33, *p* < 0.01), WHO-5 (α = 0.89; *r* = −0.47, *p* < 0.01) and GSE (α = 0.88; *r* = −0.33, *p* < 0.01).

To sum up, the newly proposed SPAS-7 is comparable with five highly discussed SPA scales and possesses good convergent and divergent validity according to Pearson’s correlation coefficient (see Table 5).

## 4. Discussion

In this study, we evaluated a number of debated versions of the SPAS that have been tested in many countries, including the original SPAS-12 [1], SPAS-9 [33], SPAS-8 [39], SPAS-7a [34] and SPAS-7b [41]. However, none of these versions showed good psychometric properties in our Chinese samples. The CFA findings illustrate that that neither the original version of the SPAS (χ^2^/df = 8.56; RMSEA = 0.167; SRMR = 0.103) nor Martin’s 9-item version (χ^2^/df = 5.96; RMSEA = 0.135) were robust in our sample. In Table 2, SPAS-8, SPAS-7a and SPAS-7b showed acceptable model fit with the data from Study 1 (*n* = 273) and the combined data from Studies 1 and 2 (*n* = 466). However, the factor loading of item 3 was far below the acceptable range for scale construction. We removed item 3 from SPAS-8 [39] to develop the 7-item version of the SPAS.

The results demonstrate that the newly developed SPAS-7, which includes items 2, 4, 6, 7, 9, 10 and 12 of the original version of SPAS proposed by [1], has good psychometric properties in Chinese samples. Based on the psychometric results, we propose a new 7-item SPAS that meets stringent criteria for good model fit (see Table 3) in both male and female models. The SPAS-7 also possesses good internal consistency, convergent validity and divergent validity.

The abnormal finding related to item 3 (I wish I wasn’t so uptight about my physique or figure) has not previously been reported in the literature. All previous discussions of the SPAS scale have classified item 3 as a positively worded item, and all have shown acceptable values for factor loading, item-total correlation and other measures. When testing factorial validity, we found that item 3 had low factor loadings for all datasets, ranging from 0.11 to 0.23 (i.e., below the cut-off value of 0.40) [78,79]. According to Merenda [72], from literature in social and behavioural science, items with factor loading less than 0.3 can be decided to be rejected. Moreover, there is no literature that reported that item 3 belongs to a special factor and its item to total correlation less than 0.4 (ranged from 0.08 to 0.15), which was considered not to be retained [80]. Cronbach’s alpha increased for all models with the removal of item 3, and the item-total correlation coefficients of item 3 were also unacceptable. SPAS-7 (without item 3) demonstrated internal consistency that was superior to other short models and correlation coefficients are comparable with other scales which indicated an acceptable concurrent validity. We also considered the dimensionality of the scale as a bimodal model [81], but this hypothesis was rejected because item 3 had a single-peaked normal distribution. After consulting the literature relating to Chinese cultural patterns, this could possibly be explained by a tendency of Chinese respondents to give positive responses in a culture that is inherently modest [50,82], which can avoid others thinking they are too arrogant [83]. This intrinsic cultural trait may explain the controversy over item 3.

Many previous studies have built models that separate data from male and female participants to eliminate the effects of gender differences. Gender differences are also an important consideration in the development of the SPAS model, and gender-specific scales were proposed [5]. Fletcher and Crocker [84] suggested that 6 of the 12 SPAS items carry a gender bias and found through item response theory analysis that items 2 and 9 (related to clothing and external evaluation) favour female subjects, whereas items 4, 6, 7, 10 (related to muscularity) favour males. We performed CFA separately on male and female groups to further evaluate SPAS-7 and SPAS-6b. The results showed that SPAS-6b only had a good model fit for the male sample, which may result from most of the items favour males and no items favour females. Hence, SPAS-7 (items 2, 4, 6, 7, 9, 10 and 12) is the most preferable scale for assessing SPA in the Chinese context.

This study also has some limitations. First, the two cross-sectional studies did not involve a wide range of respondents with different socio-economic characteristics. This may hinder the generalisability of the findings. Future studies could sample a wide range of respondents from different socio-economic backgrounds and occupational groups. Second, the gender ratio of this participant sample was unequal (male: *n* = 125; female: *n* = 341). Future research could use a stratified sampling method to ensure an appropriate distribution of gender proportions during the data collection process.

## 5. Conclusions

The current lack of a validated scale for SPA in the Chinese population may hinder the measurement of SPA levels in this population in epidemiological studies. The findings of this study provide empirical evidence to show that a new 7-item unidimensional SPAS scale, including items 2, 4, 6, 7, 9, 10 and 12 of the original SPAS, has good psychometric properties for measuring SPA in the Chinese population.

## Figures and Tables

**Table 1:** Participant demographic characteristics.

Variable	Respondents (*n* = 466)
Age mean (SD)	
Study 1	21.2 (2.21)
Study 2	23.5 (0.87)
Gender *n* (%)	
Study 1 Male	70 (25.6%)
Female	203 (74.4%)
Study 2 Male	55 (28.5%)
Female	138 (71.5%)

**Table 2:** Confirmatory Factor Analysis of the five versions of SPAS.

Model	χ^2^	Df	χ^2^/df	RMSEA [90% CI]	CFI	TLI	SRMR
Study 1 (*n* = 273)							
SPAS-12 [1]	462.202	54	8.56	0.167 [0.153–0.181]	0.964	0.956	0.103
SPAS-9 [33]	160.815	27	5.96	0.135 [0.115–0.155]	0.981	0.975	0.075
SPAS-8 [39]	27.574	20	1.38	0.037 [0.000–0.068]	0.999	0.998	0.039
SPAS-7a [34]	25.945	14	1.85	0.056 [0.019–0.089]	0.997	0.996	0.043
SPAS-7b [41]	15.848	14	1.13	0.022 [0.000–0.065]	0.999	0.999	0.031
Combo (*n* = 466)							
SPAS-12 [1]	623.530	54	11.55	0.151 [0.140–0.161]	0.964	0.956	0.094
SPAS-9 [33]	230.778	27	8.55	0.127 [0.113–0.143]	0.977	0.970	0.070
SPAS-8 [39]	37.591	20	1.88	0.043 [0.021–0.065]	0.998	0.997	0.036
SPAS-7a [34]	34.131	14	2.44	0.058 [0.035–0.082]	0.996	0.994	0.040
SPAS-7b [41]	27.652	14	1.98	0.046 [0.019–0.071]	0.998	0.997	0.033

Note. RMSEA = root mean square error of approximation, CFI = comparative fit index, TLI = Tucker–Lewis index, SRMR = standardized root mean square residual, Combo = Study 1 plus Study 2.

**Table 3:** Confirmatory factor analysis of newly proposed 7-item SPAS.

Dataset	χ^2^	Df	χ^2^/df	RMSEA [90% CI]	CFI	TLI	SRMR
Study 1 (*n* = 273)	13.623	14	0.97	0.000 [0.000–0.057]	0.999	0.999	0.035
Combo (*n* = 466)	21.990	14	1.57	0.035 [0.000–0.062]	0.999	0.998	0.032
Male (*n* = 125)	11.285	14	0.81	0.000 [0.000–0.071]	0.999	0.999	0.038
Female (*n* = 341)	35.635	14	2.55	0.067 [0.040–0.095]	0.996	0.993	0.045

Note. RMSEA = root mean square error of approximation, CFI = comparative fit index, TLI = Tucker–Lewis index, SRMR = standardized root mean square residual. Combo = Study 1 plus Study 2.

**Table 4:** Descriptive statistics and correlations for the items of SPAS-7 in Studies 1 and 2.

	Study 1							Study 2						
Item	(1)	(2)	(3)	(4)	(5)	(6)	(7)	(1)	(2)	(3)	(4)	(5)	(6)	(7)
(1) SPAS2	-							-						
(2) SPAS4	0.46 **	-						0.41 **	-					
(3) SPAS6	0.43 **	0.67 **	-					0.40 **	0.55 **	-				
(4) SPAS7	0.47 **	0.70 **	0.70 **	-				0.49 **	0.59 **	0.64 **	-			
(5) SPAS9	0.35 **	0.62 **	0.55 **	0.54 **	-			0.39 **	0.48 **	0.42 **	0.46 **	-		
(6) SPAS10	0.30 **	0.50 **	0.55 **	0.56 **	0.55 **	-		0.33 **	0.45 **	0.39 **	0.47 **	0.51 **	-	
(7) SPAS12	0.47 **	0.60 **	0.58 **	0.58 **	0.51 **	0.55 **	-	0.39 **	0.47 **	0.44 **	0.50 **	0.41 **	0.46 **	-
M	3.3	3.5	3.4	3.1	3.6	3.6	3.5	3.6	3.7	3.5	3.1	3.8	3.9	3.5
SD	1.2	1.1	1.1	1.2	1.1	1.1	1.2	1.2	1.2	1.1	1.1	1.1	1.0	1.2
r*_it_*	0.52	0.77	0.75	0.77	0.66	0.63	0.70	0.53	0.67	0.64	0.82	0.60	0.58	0.60
α*_iid_*	0.89	0.86	0.86	0.86	0.88	0.88	0.87	0.85	0.83	0.83	0.82	0.84	0.84	0.84
Skewness	−0.48	−0.59	−0.44	−0.19	−0.66	−0.48	−0.44	−0.71	−0.80	−0.70	−0.35	−0.80	−0.85	−0.56
Kurtosis	−0.72	−0.48	−0.62	−0.85	−0.25	−0.44	−0.67	−0.48	−0.26	−0.35	−0.77	−0.09	0.14	−0.68

Note. **. Correlation is significant at the 0.01 level (2-tailed), r*_it_* = Corrected item-total correlations, α*_iid_* = Cronbach’s alpha, if item deleted.

**Table 5:** Correlation for six SPAS models in relation to construct-related scales in Study 2.

Scale	SPAS-12	SPAS-9	SPAS-8	SPAS-7a	SPAS-7b	SPAS-7
	α = 0.88	α = 0.84	α = 0.83	α = 0.80	α = 0.82	α = 0.86
	ω = 0.88	ω = 0.85	ω = 0.84	ω = 0.80	ω = 0.82	ω = 0.86
SPAS-12	1					
SPAS-9	0.97 **	1				
SPAS-8	0.96 **	0.98 **	1			
SPAS-7a	0.94 **	0.98 **	0.97 **	1		
SPAS-7b	0.92 **	0.97 **	0.99 **	0.97 **	1	
SPAS-7	0.96 **	0.97 **	0.99 **	0.95 **	0.97 **	1
Divergent Validity						
SWLS	−0.40 **	−0.36 **	−0.35 **	−0.35 **	−0.31 **	−0.36 **
RSE	−0.38 **	−0.33 **	−0.33 **	−0.32 **	−0.30 **	−0.33 **
WHO-5	−0.54 **	−0.49 **	−0.48 **	−0.47 **	−0.44 **	−0.47 **
GSE	−0.38 **	−0.35 **	−0.33 **	−0.33 **	−0.31 **	−0.33 **
Convergent Validity						
SIAS-6	0.53 **	0.55 **	0.55 **	0.52 **	0.54 **	0.55 **

Note. **. Correlation is significant at the 0.01 level (2-tailed).

## Data Availability

The dataset used and/or analysed in this study is available from the corresponding author on reasonable request.
